# Mutual information based stock networks and portfolio selection for intraday traders using high frequency data: An Indian market case study

**DOI:** 10.1371/journal.pone.0221910

**Published:** 2019-08-29

**Authors:** Charu Sharma, Amber Habib

**Affiliations:** Department of Mathematics, Shiv Nadar University, Gautam Buddha Nagar, Uttar Pradesh, India; The Bucharest University of Economic Studies, ROMANIA

## Abstract

In this paper, we explore the problem of establishing a network among the stocks of a market at high frequency level and give an application to program trading. Our work uses high frequency data from the National Stock Exchange, India, for the year 2014. To begin, we analyse the spectrum of the correlation matrix to establish the presence of linear relations amongst the stock returns. A comparison of correlations with pairwise mutual information shows the further existence of non-linear relations which are not captured by correlation. We also see that the non-linear relations are more pronounced at the high frequency level in comparison to the daily returns used in earlier work. We provide two applications of this approach. First, we construct minimal spanning trees for the stock network based on mutual information and study their topology. The year 2014 saw the conduct of general elections in India and the data allows us to explore their impact on aspects of the network, such as the scale-free property and sectorial clusters. Second, having established the presence of non-linear relations, we would like to be able to exploit them. Previous authors have suggested that peripheral stocks in the network would make good proxies for the Markowitz portfolio but with a much smaller number of stocks. We show that peripheral stocks selected using mutual information perform significantly better than ones selected using correlation.

## 1) Introduction

High frequency trading is the buying and selling of large numbers of stocks in very short intervals of time, usually fractions of seconds. With advancement in computing and technology, it is possible for investors to carry out such trades using algorithmic trading. A good trading strategy should be equipped to understand the movement of stocks even at a tick-by-tick level. Among various factors, which influence the change in a stock price, change in the prices of other stocks is one of the most significant. Over the years, many researchers [1–11] have used random matrix theory (RMT) on the empirical correlation matrix to understand the co-movements of the stocks based on daily rates of return. The cross-correlation matrix of daily stock returns with respect to a developed market like US was studied in depth using RMT in 2001 [2]. Later, in 2007, Pan & Sinha [4] studied the cross correlation matrix of daily stock returns both in developed and developing countries, namely US and India. Under all these studies, spectral properties of empirical correlation matrix of rate of returns were compared to that of random matrix which would have appeared if stock returns were uncorrelated [2,4]. It was observed that an emerging market like India shows stronger interactions in price movements as compared to a developed market. Their analysis was also based on the daily rates of return. In this paper, we first study the spectrum of the correlation matrix but now at high frequency level, using 30-second time intervals, with respect to the Indian market. Most of the studies based on RMT performed on different exchanges in the past suggest that the bulk of the eigenvalues are in agreement with the Marchenko-Pastur distributions with few exceptions. The large eigenvalues which deviate from the Marchenko-Pastur distributions are studied to understand the influence of the market as a whole and also sectorial effects [1–11]. However, correlation coefficient is a measure of linear relation between the variables. If there is a non-linear relationship, the correlation coefficient may fail to capture it. Thus, there is a great need to develop methods which are able to detect and describe the non-linearity amongst the stocks, especially at a high frequency level.

Mutual information is one such measure that measures mutual dependence between random variables in general. Researchers in the past have used methods based on mutual information in building biological networks [12–13]. However, developing stock networks based on mutual information is at a very early stage [14–23]. In 2018, Guo, Zhang and Tian [18] studied Chinese stock network using the mutual information networks based on daily returns of stocks. They showed that mutual information based model captures the daily dynamics of the stock networks better in comparison to the correlation based model. However, exploration of this method at a high frequency level was left as an open problem. In 2019, Barbi and Prataviera [20] studied networks based on nonlinear dependencies in Brazilian equity market with a tick size of 15 minutes. In the same year, Khoojine and Han [21] studied Chinese stock market turbulence in the year 2015–2016 by analysing topological properties of the complex network obtained based on mutual information. We, in our analysis have considered high frequency data of Indian stock market, with a tick size of 30 seconds. We compare the traditional correlation coefficient method with the mutual information method and show that the mutual information method is a more efficient method at a high frequency level as well. Centrality measures like eigenvector centrality help us to analyse important stocks and business sectors in respect to the Indian stock market.

We also demonstrate how networks based on mutual information can be used for picking portfolios with small number of stocks, preferred by the intraday traders. The Markowitz or mean-variance model for portfolio selection is the traditional model used for selecting efficient portfolios. This method uses variance of the portfolio as a quantifier of the risk associated with the portfolio and tries to minimize it, while at the same time attaining required return from the portfolio. This method works well under the assumption of a linear relation amongst the stocks. To overcome this issue, Philippatos and Wilson [23] gave an entropy-based model which minimizes the entropy of the portfolio and maximizes the rate of return. Since then many researchers have proposed different models based on entropy to get a diversified portfolio, which is less risky, and at the same time give good returns [24]. These strategies are useful for long-term investors but might not work for a short-term investor like a day trader who cannot afford to invest in too many stocks at a time. In 2013, Pozzi, Matteo and Aste [25], suggested that stocks present in the periphery of a minimum spanning tree network based on exponential weighted correlations are a good choice of investment. In our analysis, we compare the peripheral portfolios obtained using correlations and mutual information methods and show that portfolios based on mutual information techniques are more efficient.

Estimating mutual information with good accuracy is itself an important research area in the field of information theory. In the past, various numerical algorithms have been proposed to estimate mutual information accurately and efficiently [26–28]. Cellucci, Albano and Rapp [26] compared some of these algorithms in terms of efficiency and accuracy. Their analysis showed that the Fraser-Swinney algorithm is the best in terms of accuracy but takes quite a long time. The adaptive partition method takes about 0.5% of the calculation time required by Fraser-Swinney while its accuracy is better in comparison to the uniform bin method. We use the adaptive partition method to estimate mutual information on 30-second data.

The remaining part of the paper is broadly divided into 4 sections. In section 2, we describe the data used in our analysis. In section 3, we give an overview of the methods and methodology used. In section 4, we give a detailed comparative study of the networks based on their topological features. In this section, we also give an application on how to use the dynamics of stock networks for portfolio selection especially for an intraday trader. In the last section, we conclude by highlighting the salient observations and interpretations that follow from our analysis.

## 2) Data description

We obtained tick-by-tick data for the year 2014 from the National Stock Exchange, India. The data was filtered to get all the stocks listed in CNX100 during that year. 11 stocks were dropped from the analysis due to insufficient or missing data. Table 1 gives the details of the composition of the CNX100 index. The exchange opens at 9 o’clock in the morning and is functional till 4 PM. The trades start picking up in the first half-hour, while the last half-hour shows some ambiguity or incompleteness in the data. Considering this, we have used the data between 9:30AM and 3:30PM in our analysis. Every 30-second interval is considered a tick, and thus in each day we have 720 tick points for each stock. For the *kth* stock, we first calculate the volume weighted average price (VWAP), ${\hat{S}}_{t}^{k}$ per 30 seconds,

$${\hat{S}}_{t}^{k}=\frac{\sum_{i}v_{i}^{k}S_{i}^{k}}{\sum_{i}v_{i}^{k}}$$ (1)

Here, $v_{i}^{k}$ is the volume of the *kth* stock traded at the actual tick *i* and $S_{i}^{k}$ is the stock price at the tick *i* in 30- second widow at time t. The log return at time t is then calculated using Eq 2. The gaps, where no trades were seen during a 30-second interval, were filled with the last traded price of the stock.

$$R_{t+1}^{k}=\ln\left({\hat{S}}_{t+1}^{k}\right)-ln({\hat{S}}_{t}^{k})$$ (2)

**Table 1:** Sectorial distributions of the 89 stocks considered in our analysis.

Industry Type	No. of Stocks analysed
INDUSTRIAL MANUFACTURING	5
CEMENT & CEMENT PRODUCTS	5
SERVICES	2
AUTOMOBILE	10
CONSUMER GOODS	14
PHARMA	10
FINANCIAL SERVICES	14
ENERGY	10
TELECOM	3
METALS	6
CONSTRUCTION	2
IT	6
CHEMICALS	1
FERTILISERS & PESTICIDES	1

In addition, we considered the year 2014 for our analysis since this was a year when general elections were held in India and a change in government was observed. We wanted to analyse the effect of this major event on the network. For this purpose, we divided our data into three parts: (a) pre-election period: Jan-Feb 2014 (b) election period: Mar-May 2014, (c) post-election period Jun-Dec 2014. Since promotional rallies took place in the month of March, elections in the month of April and declaration of results in the month of May, so we marked the months March, April and May as the election period. Table 2 summarizes the details of the data. It was observed, that the market became more volatile during the time when election results were announced (S1(B) Fig) and this trend continued for some time during the post-election period (S1(C) Fig).

**Table 2:** Summary of three different datasets, pre-election, election, post-election.

	Jan-Feb 2014	Mar-May 2014	Jun-Dec 2014
No. of trading days	42	46	141
No. of stocks	89	89	89
No. of samples (30 sec tick size)	30198	33074	101379
average rate of return ($\bar{r}$)	−1.80×10^−6^	−2.81×10^−7^	−1.13×10^−6^
sample standard deviation(*s*)	1.31×10^−4^	1.73×10^−4^	1.57×10^−4^
maximum rate of return	1.04×10^−3^	2.41×10^−3^	1.65×10^−3^
minimum rate of return	−2.86×10^−3^	−2.43×10^−3^	−2.26×10^−3^
range	3.9×10^−3^	4.83×10^−3^	3.92×10^−3^

## 3) Methods and methodology

### 3.1 RMT approach on correlation coefficient matrix

**Fig 1:**
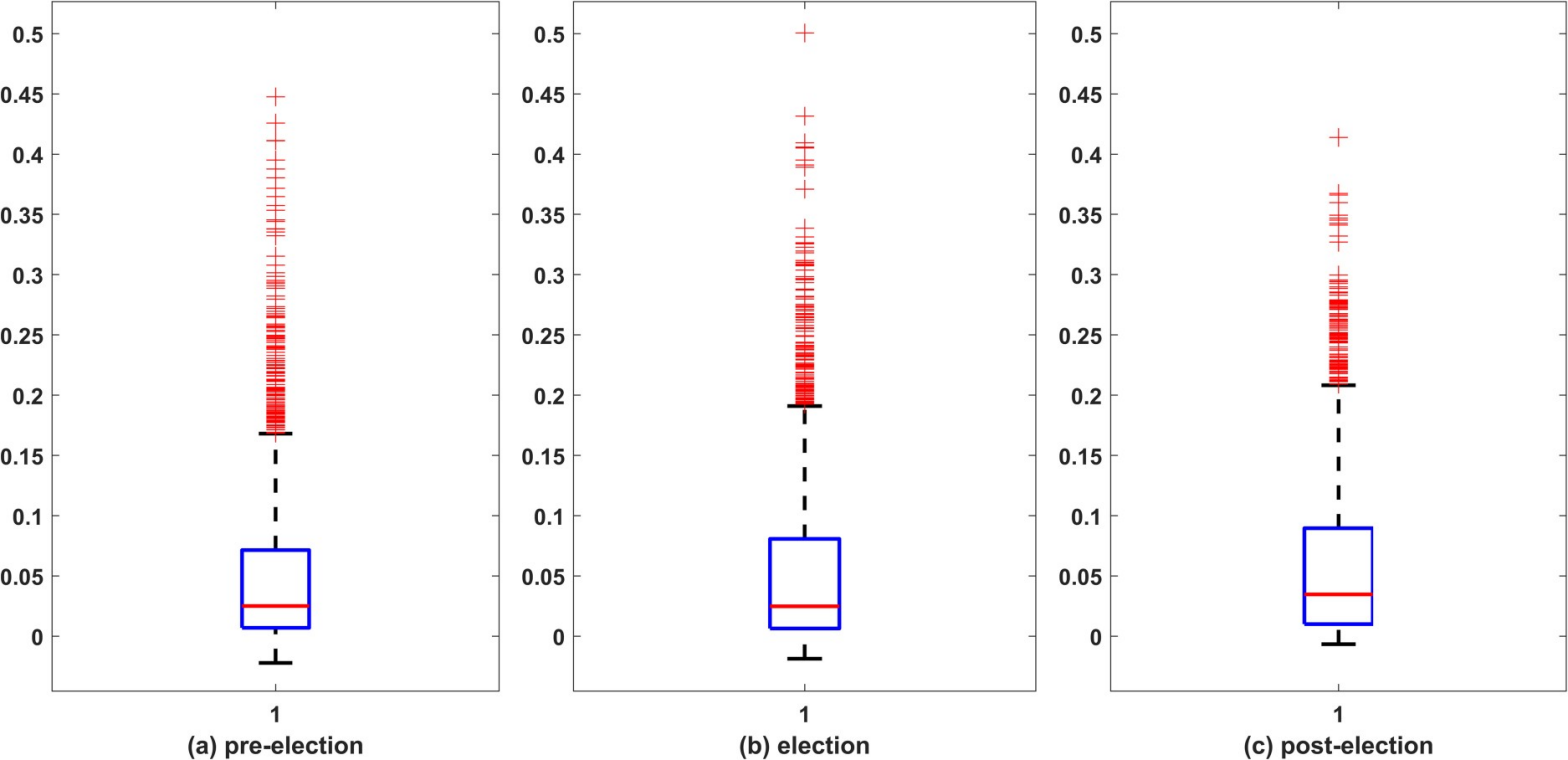
Box plot representation of distribution of correlation coefficient between all 3916 pairs of stocks. (a) corresponds to pre-election period, (b) election period and (c) post-election period; the central red line in the box indicates the median, and the bottom and top edges of the box indicate the 25th and 75th percentiles, respectively. Outliers are represented by +. The 75th percentile corresponding to election period and post-election is high in comparison to pre-election period. Also more outliers (+) are visible during election period, which accounts for the distinction of correlation patterns in the three time-periods.

Correlation coefficient between two random variables measures the strength of the linear relationship between them. We build the correlation matrix *C* of size 89×89 separately for all the three time spans, pre-election, election and post-election. Fig 1 gives the boxplot corresponding to the distribution of the correlation coefficients for the three different time spans. During the election period, more pairs are seen to have higher correlation coefficient in comparison to pre-election and post-election periods. This can be understood as a result of the election effect on the market as a whole. Researchers in the past have studied the effect of general elections on the stock market [29] and observed that during election time investors develop common expectations around the anticipated results. Thus, during the election process, market becomes volatile and at the same time stronger and more number of interactions are observed amongst the stocks.

The statistical properties of the correlation matrix are well established in the literature [1–11]. With a large number of variables say *k* and a large number of sample points say *m*, and under the hypothesis that *C* is a random correlation matrix, the distribution of eigenvalues of *C* can be well approximated by the Marchenko-Pastur distribution. For large *k* and *m*, i.e., *k*→∞, *m*→∞ such that $Q=\frac{m}{k}$ is fixed, the probability distribution of the eigenvalues of a random correlation matrix is given by

$$f_{rm}(\lambda )=\frac{Q}{2\pi }\frac{\sqrt{\left(\lambda_{max}-\lambda )(\lambda -\lambda_{min}\right)}}{\lambda }$$ (3)

where *λ*_*max*_ and *λ*_min_ are the maximum and minimum eigenvalue of *C* given by

$$\lambda_{max}={\left(1+\sqrt{\frac{1}{Q}}\right)}^{2}\;\mathrm{and}\;\lambda_{min}={\left(1-\sqrt{\frac{1}{Q}}\right)}^{2}$$ (4)

If the empirical distribution based on sample data deviates from the theoretical distribution, then one can reject the null hypothesis that entries in *C* are random. S2 Fig gives the empirical pdfs and theoretical pdfs of the eigenvalues of *C*. In addition, Table 3 summarizes the statistics from the empirical and theoretical distribution. More than 40% of the eigenvalues of the correlation matrix were seen to be deviated from the Marchenko-Pastur distribution in all three time-spans. Eigenvectors corresponding to large eigenvalues, also known as principal components, carry useful information in comparison to eigenvectors corresponding to small eigenvalues. We therefore analysed only the eigenvectors corresponding to large eigenvalues deviating from the RMT.

**Table 3:** Estimated parameters corresponding to distribution of spectrum of correlation matrix.

Serial No.	Eigenvalue estimation	pre-election	election	post-election
1.	*λ*_*max*_ (theoretical)	1.11	1.11	1.06
2.	${\hat{\lambda }}_{max}$ (empirical)	7.40	7.68	8.23
3.	${\hat{\lambda }}_{max}/\lambda_{max}$	6.66	6.94	7.77
4.	${\lambda_{min}<\hat{\lambda }<\lambda }_{max}$ (%)	58.43%	50.56%	42.13%
5.	${\hat{\lambda }>\lambda }_{max}$ (%)	6.74%	8.99%	7.87%
6.	${\hat{\lambda }<\lambda }_{min}$ (%)	34.83%	40.45%	50.00%

*λ*_*max*_ and *λ*_*min*_ are calculated using Eq 4 and ${\hat{\lambda }}_{max}$ is maximum of the eigenvalue of the correlation matrix. Row 1 and row 2 in the table give the parameter estimations; row 3 gives the ratio; row 4 gives proportion of eigenvalues $\hat{\lambda }$ lying within theoretical limits; rows 5 and 6 give proportion of eigenvalues $\hat{\lambda }$ greater than and less than theoretical limits respectively.

Also, in order to show that the deviation from the RMT is not because of the finite number of variables, 89 in our case, we tested this procedure on the testing data generated by randomly shuffling the returns for each stock. S3 Fig gives the comparison of the empirical pdfs and theoretical pdfs of the eigenvalues of *C* corresponding to the testing data. It is quite evident that the testing data matches well with the Marchenko-Pastur distribution, indicating that the deviations from this distribution in the original data are genuinely due to the correlation between the stock returns.

### 3.2 Mutual information method

Mutual Information between two random variables captures mutual dependence between them and is zero if and only if they are independent. In information theory, Shannon Entropy is a measure of “uncertainty” or “unpredictability” of a random variable or a random vector. For discrete random variables *X* and *Y*, their joint entropy is defined as

$$H(X,Y)=-\sum_{i}\sum_{j}f_{X,Y}(x_{i},y_{j})log(f_{X,Y}(x_{i},y_{j}))=E[-log(f_{X,Y})]$$ (5)

where *f*_*X*,*Y*_(*x*_*i*_,*y*_*j*_) is the joint probability mass function of *X* and *Y*. Also, entropy of a discrete random variable with probability mass function *f*_*X*_ is defined as

$$H(X)=-\sum_{i}f_{X}(x_{i})log(f_{X}(x_{i}))=E[-log(f_{X})]$$ (6)

The mutual information of discrete random variables X and Y is defined as

$$I(X,Y)=H(X)+H(Y)-H(X,Y)=\sum_{i}\sum_{j}f_{X,Y}(x_{i},y_{j})log(\frac{f_{X,Y}(x_{i},y_{j})}{f_{X}(x_{i})f_{Y}(y_{j})})$$ (7)

A generalization to the continuous case is

$$I(X,Y)=\iint f_{X,Y}(x,y)log(\frac{f_{X,Y}(x,y)}{f_{X}(x)f_{Y}(y)})dxdy$$ (8)

Cellucci, Albano and Rapp [26] carried out a comparative study of methods to estimate mutual information in the case of continuous random variables. We have used the non-uniform adaptive partition algorithm in preference to the Fraser-Swinney algorithm [26], as it offers the best combination of efficiency and accuracy. The key idea of this algorithm is to estimate joint probability density function *f*_*X*,*Y*_ of random variables *X* and *Y* with high accuracy using a non-uniform partition of XY plane. We partition intervals (*x*_*min*_,*x*_*max*_) and (*y*_*min*_,*y*_*max*_) using *N*_*E*_ elements such that there are approximately equal number of sample points in each element of a partition; *x*_*min*_ and *y*_*min*_ are the respective empirical minimum of *X* and *Y* and similarly *x*_*max*_ and *y*_*max*_ are the respective maximums. Also *N*_*E*_ is calculated such that in case of independent random variables, the expected number of elements in each partition of XY plane should at least be 5 which is equivalent to find greatest integer *N*_*E*_ such that $N_{E}\leq {\left(\frac{N_{D}}{5}\right)}^{2}$, where *N*_*D*_ is total number of sample points [26]. Table 2 gives the number of sample points (*N*_*D*_) in different time-periods.

Based on mutual information, the normalized distance [18] between two stocks *k* and *s* is defined as

$$d(R^{k},R^{s})=1-\frac{I(R^{k},R^{s})}{H(R^{k},R^{s})}$$ (9)

where *R*^*k*^ and *R*^*s*^ random vectors representing the rate of returns of stock *k* and *s* respectively.

### 3.3 Minimum spanning tree

For connected graphs, a spanning tree is a subgraph that connects every node in the graph and has no cycles. There may exist more than one spanning tree for a given graph. If weights are assigned to each edge, then a minimum spanning tree (MST) is a spanning tree whose edges have the least total weight. To build a MST stock network, we quantify the distance between each pair of stocks and use this distance as the edge weight between each pair of stocks. In our analysis, we have considered two models on stocks rate of returns, one based on linear relationship using correlation coefficient and the other based on non-linear relationship using mutual information. For both cases, we define measure of distance between pairs of stocks, Eqs 9 and 10, and use them to construct MSTs. There are two well-known methods to construct MST: Kruskal’s algorithm and Prim’s algorithm. Kruskal’s algorithm is more suited to the case when we are working on a sparse network i.e. number of edges are less while Prim’s algorithm is more suited when we have a dense graph. Since stock networks are dense, we chose Prim’s algorithm to build the MST network. Prim’s algorithm starts with an arbitrary node; add an edge from this node to another node corresponding to the shortest distance, which has not yet been included in the graph. Then repeat this process until all the nodes are included in the graph.

## 4) Discussion

### 4.1 Comparative study of the methods

In the past, researchers have used correlation coefficient of daily rate of return of stocks to understand the networks amongst them [1–11]. Pan and Sinha [4] studied daily rate of return in context of Indian stock market and they observed strong correlation movement in comparison to a developed country like US. In their study, they observed that the bulk of the data is in synergy with RMT with a few deviations that indicate market effect. In our analysis, we work on high frequency data with a tick size of 30 seconds. Around 42%, 50% and 58% deviations are observed from the RMT during pre-election, election and post-election period respectively, out of which 6.74%, 8.99% and 7.87% deviations correspond to large eigenvalues for the three time spans respectively (Table 3). The compositions of the first and second eigenvectors (S4 Fig) indicate that the stocks corresponding to financial sector and the IT sectors are the key contributors over all the three-time spans. Unlike earlier studies based on daily rate of returns [2, 4], the composition of the first eigenvector in our analysis points towards a sectorial effect. During the pre-election time, financial sector, IT sector and energy sector are key contributors in the second eigenvector. However, during the election and post- election it is the financial sector and the energy sector, which are the dominant contributors towards second eigenvector. From third eigenvector onwards, no direct interpretation is observed.

**Fig 2:**
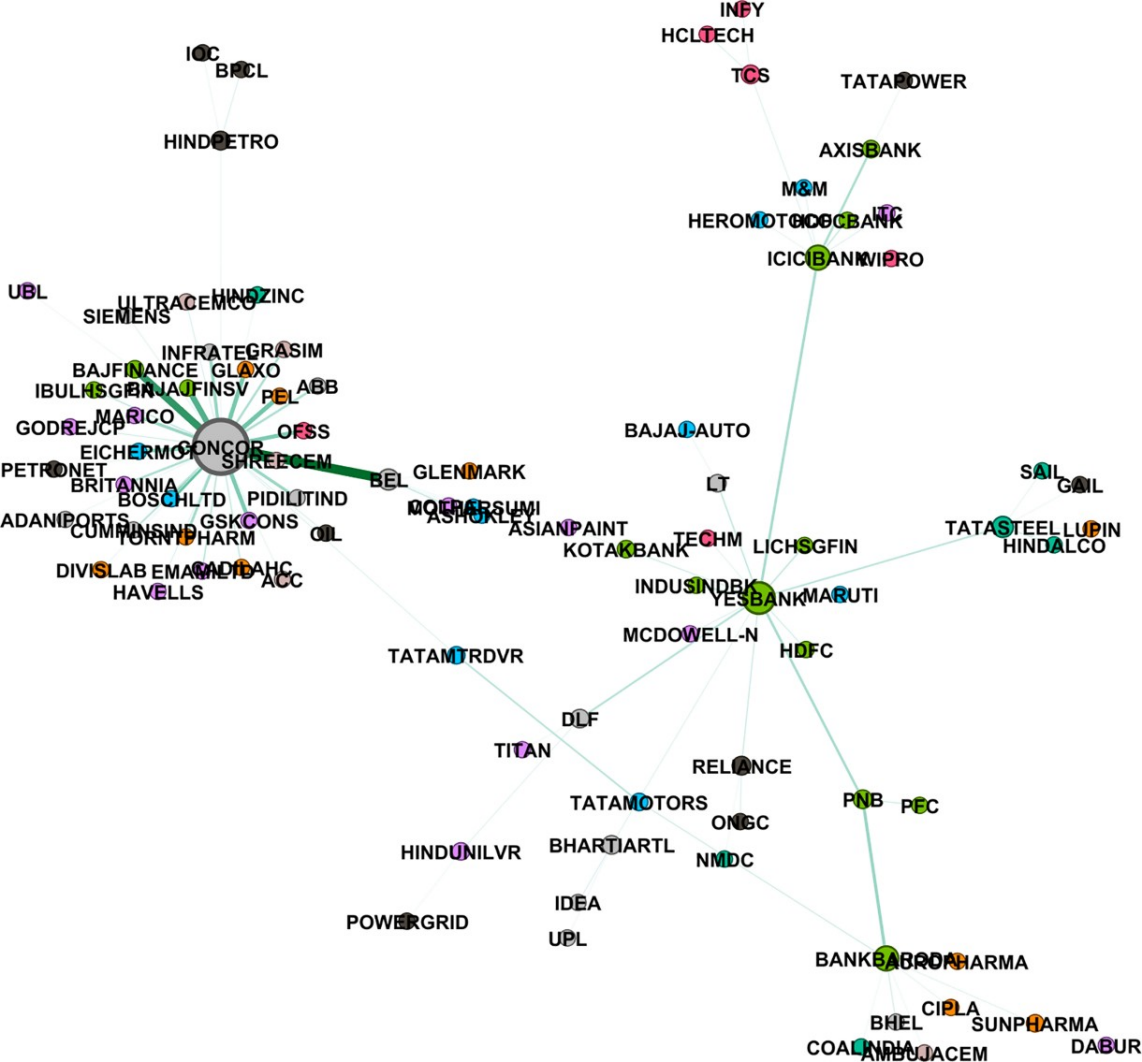
Mutual information based MST network, pre-election period (Jan-Feb 2014). Different colours represent different sectors, also size of a node is proportional to the degree of the node and width of the edge is inversely proportional to the distance between two nodes.

**Fig 3:**
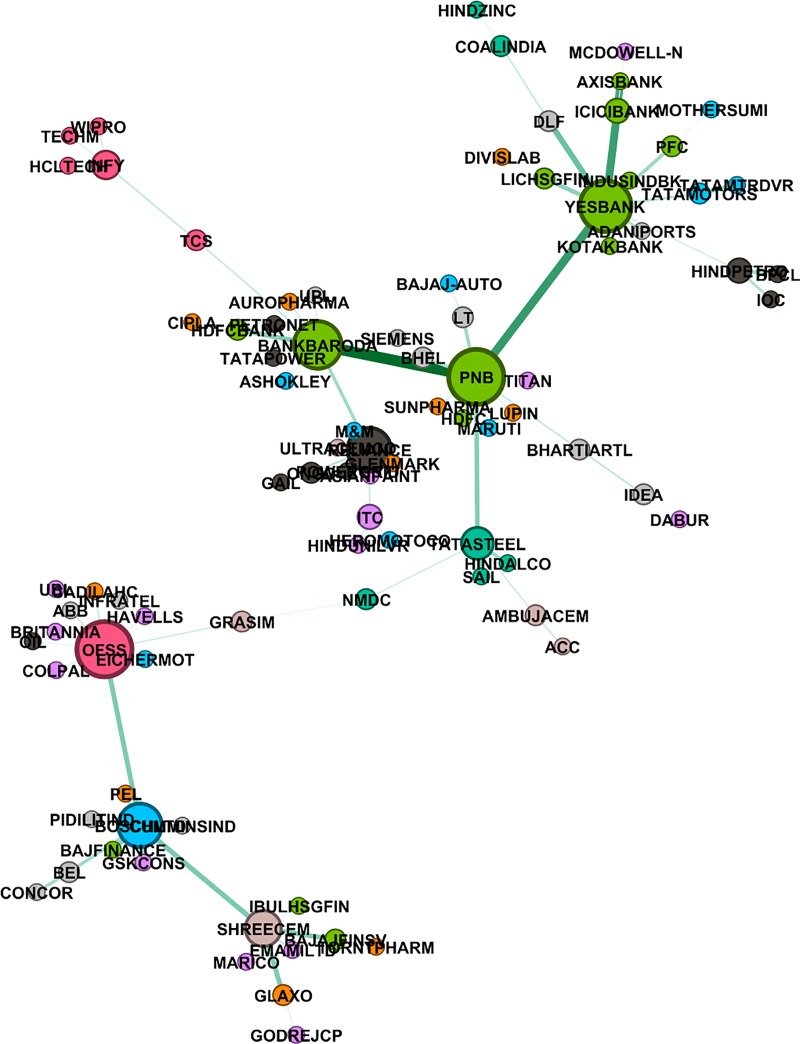
Mutual information based MST network, election period (Mar-May 2014). Different colours represent different sectors, also size of a node is proportional to the degree of the node and width of the edge is inversely proportional to the distance between two nodes.

**Fig 4:**
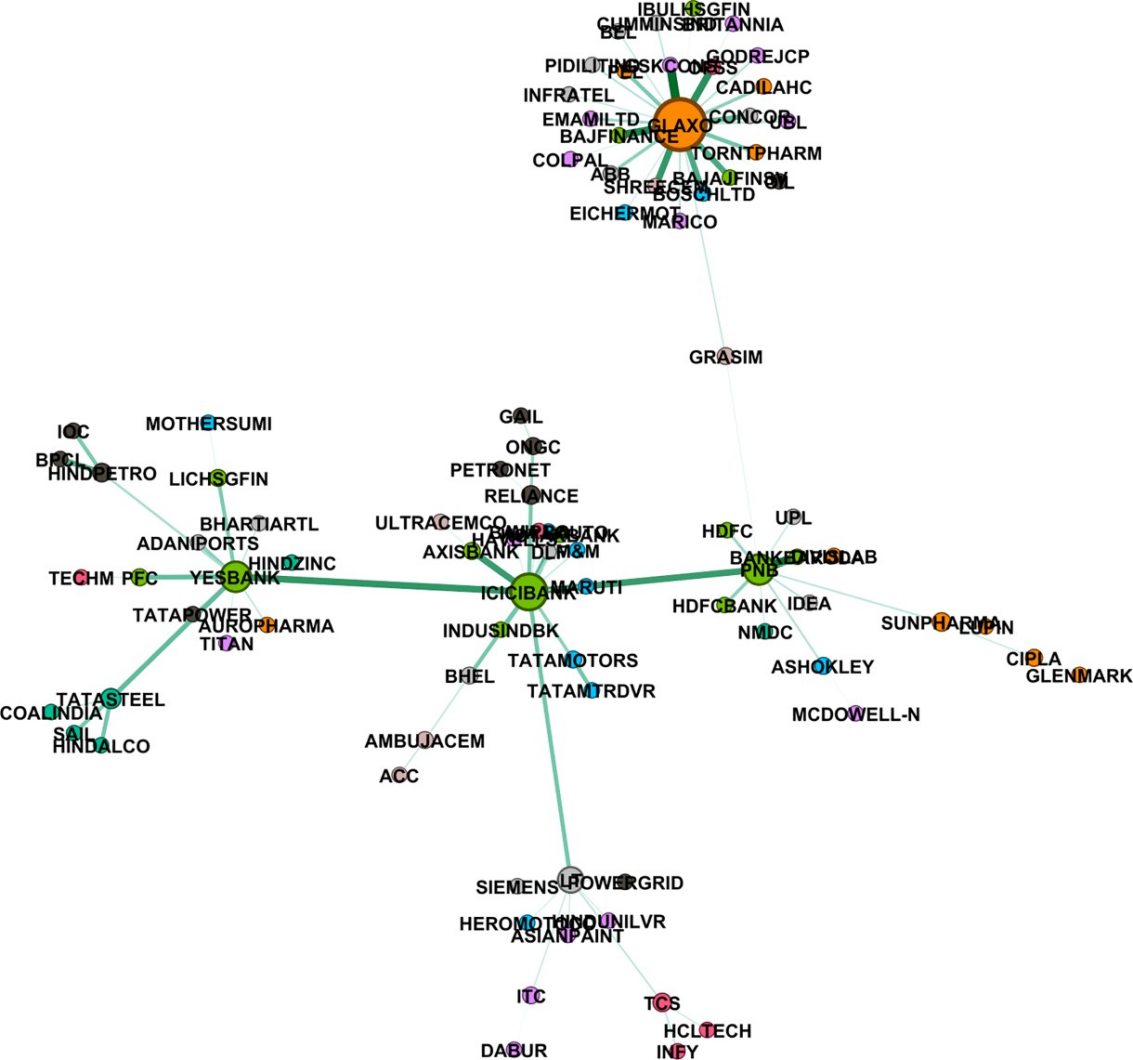
Mutual information based MST network, post-election period (Jun-Dec 2014). Different colours represent different sectors, also size of a node is proportional to the degree of the node and width of the edge is inversely proportional to the distance between two nodes.

Deviations from the RMT suggest that the correlations observed are not all due to randomness and hence, in order to study the linear relationship between the stocks in depth, we construct a minimum spanning tree graph with the distance metric as,

$$d(R^{k},R^{s})=\left(1-|\rho_{R^{k},R^{s}}|\right),$$ (10)

where $\rho_{R^{k},R^{s}}$ is correlation coefficient between the rate of returns of the two stocks *R*^*k*^,*R*^*s*^. To capture the non-linearity in the data, we also construct the mutual information based MST using the distance given in Eq 9. We have used *Gephi 0*.*9*.*2* (https://gephi.org/) to plot these networks. We also did a hypothesis test of independence at 5% level of significance. Null hypothesis: two stocks are independent; Alternate hypothesis: they are not independent. In the cases where null hypothesis cannot be rejected, we assign the value of mutual information to be zero. Figs 2–4 give networks based on mutual information for the pre-election, election and post-election periods respectively. Similar networks based on correlation method are provided as supplementary figures S5–S7 Figs.

**Fig 5:**
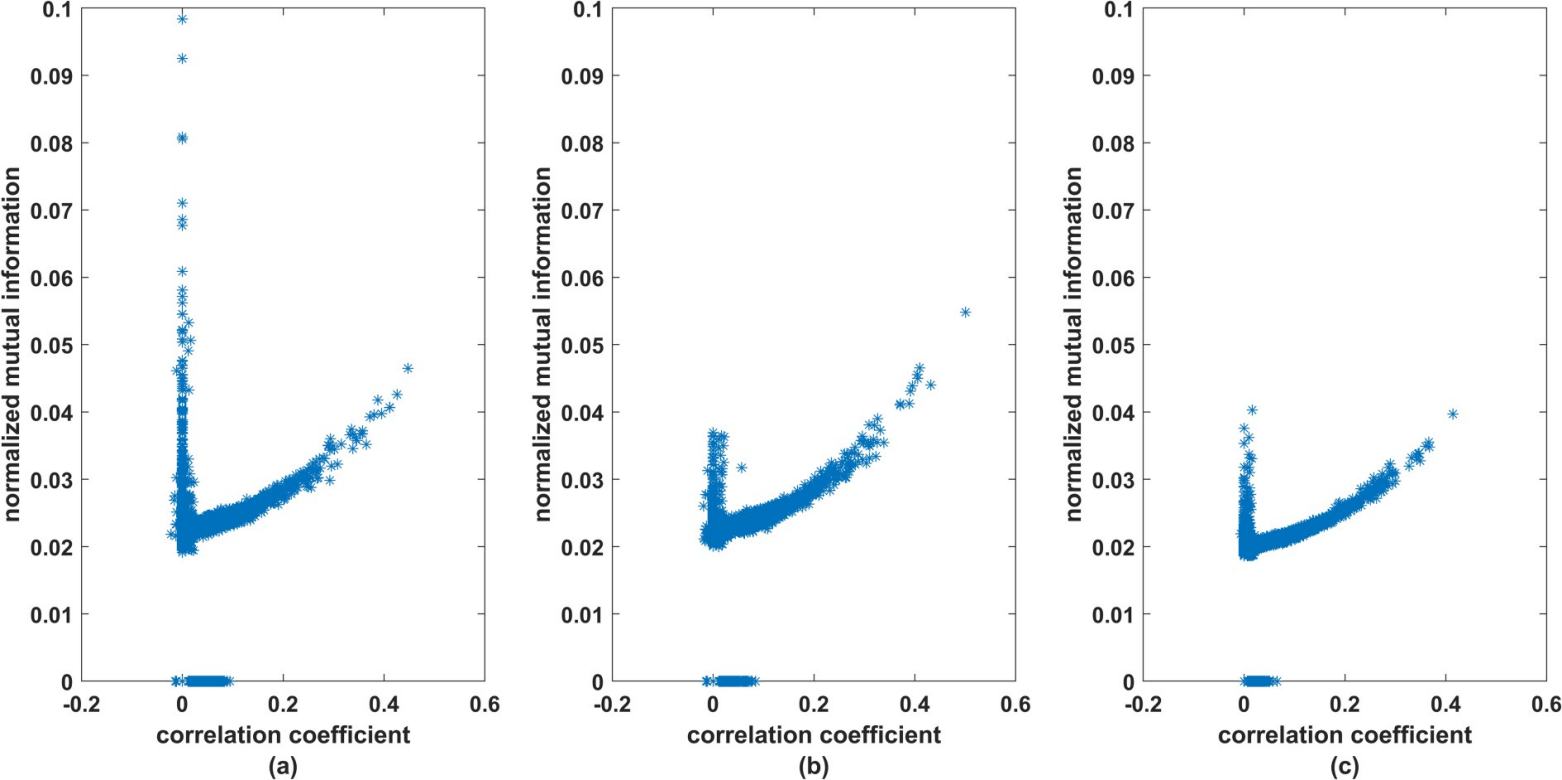
Correlation coefficient versus mutual information: high frequency data. On x-axis we have correlation coefficients and on y-axis we have normalized mutual information for all 3916 pairs. (a), (b) and (c) correspond to pre-election, election and post-election periods.

**Fig 6:**
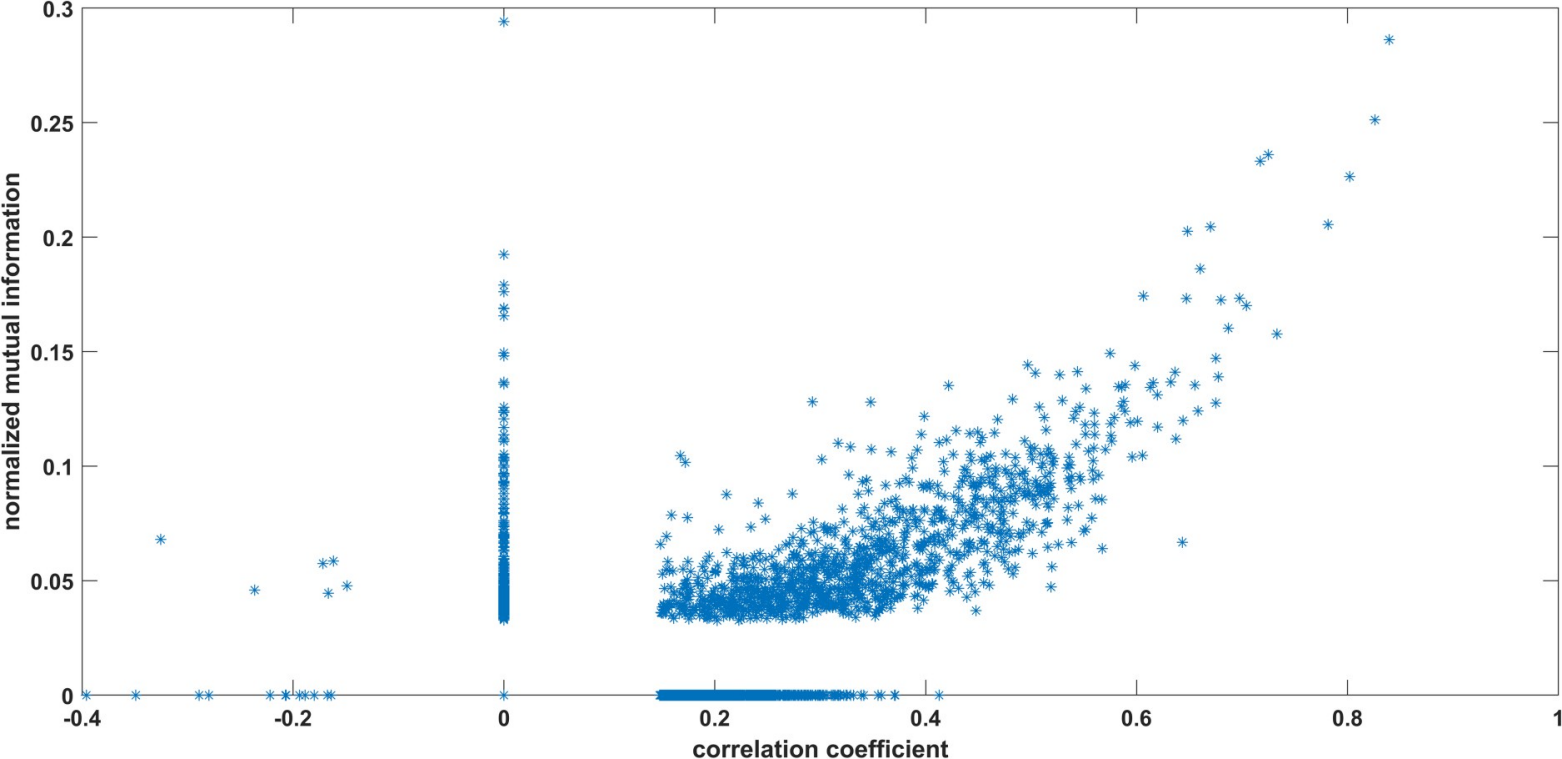
Correlation coefficient versus mutual information: daily returns for the year 2014. On x-axis we have correlation coefficients and on y-axis we have normalized mutual information for all 3916 pairs. (a), (b) and (c) correspond to pre-election, election and post-election periods.

In order to analyse how effective is the non-linear method based on mutual information in comparison to the linear method based on correlation coefficient, we plot the normalized mutual information values against the respective correlation coefficient values of all the 3916 pairs of stocks. Normalized mutual information between two random variables *X* and *Y* is defined as

$$U(X,Y)=2\frac{I(X,Y)}{H(X)+H(Y)}$$ (11)

where *I*(*X*,*Y*) is mutual information between two random variables and *H*[*X*],*H*[*Y*] are their respective entropy. Fig 5 gives the plots corresponding to all three time spans. We observe that in all three cases, larger values of correlation coefficient are associated with larger values of normalized mutual information but there are substantial number of instances when smaller values of correlation coefficient are associated with large values of mutual information. This suggests that the non-linear method based on mutual information not only managed to capture strong linear relationships but at the same time capture the non-linearity found in the data which the method based on correlation coefficient failed to capture. Also, there are instances when mutual information values are small in comparison to the values of correlation. However, such instances are few and also the magnitude of the correlation coefficient of the pairs falling in this category is very small, less than 0.1. We believe this could be due to some random noise in the data. Overall, it is quite evident that mutual information method is more effective in capturing the interaction between the stocks, in comparison to the widely used correlation method for building stock networks. We also plot similar graph between correlation and mutual information but now considering daily rate of returns for the entire year 2014 (Fig 6). It is observed from the graph that except for one pair, high values of mutual information are associated with high values of correlations. There are some pairs where higher values of mutual information are associated with lower values of correlations but the magnitude of the mutual information in such cases are not very large. It is observed, that the non-linearity captured by the mutual information at high frequency level is much more pronounced in comparison to daily return data.

In order to get more insights, we also study some of the centrality measures like degree, degree distributions and eigenvector centrality with respect to stock networks obtained using mutual information and correlation coefficient methods. We use the eigenvector centrality measure to pick the best stocks for asset allocation at each instant of a tick and study the stability of their selection. We also compare the performance of the portfolio selected based on mutual information method with the portfolio selected based on correlation method and the portfolio selected using Markowitz method.

### 4.2 Degree distribution of the networks

In a graph, the degree of a node is the number of links attached to that node. Nodes with high degrees are important and they are called hubs. Stocks corresponding to hubs in a network can be viewed as stocks, which are linked to good enough number of other stocks, and thus any flow of information amongst the stocks found in periphery of hubs takes place through these hubs. We looked at degree distribution of the stocks in all the three MST stock networks. Stocks from financial sector, like ICICI Bank, PNB, Yes Bank, observed to have degrees more than 4, are clearly the dominant stocks irrespective of the methods and the time span. Other than financial sector, there are a few stocks from the IT and Energy sector which are found to have high degree.

**Fig 7:**
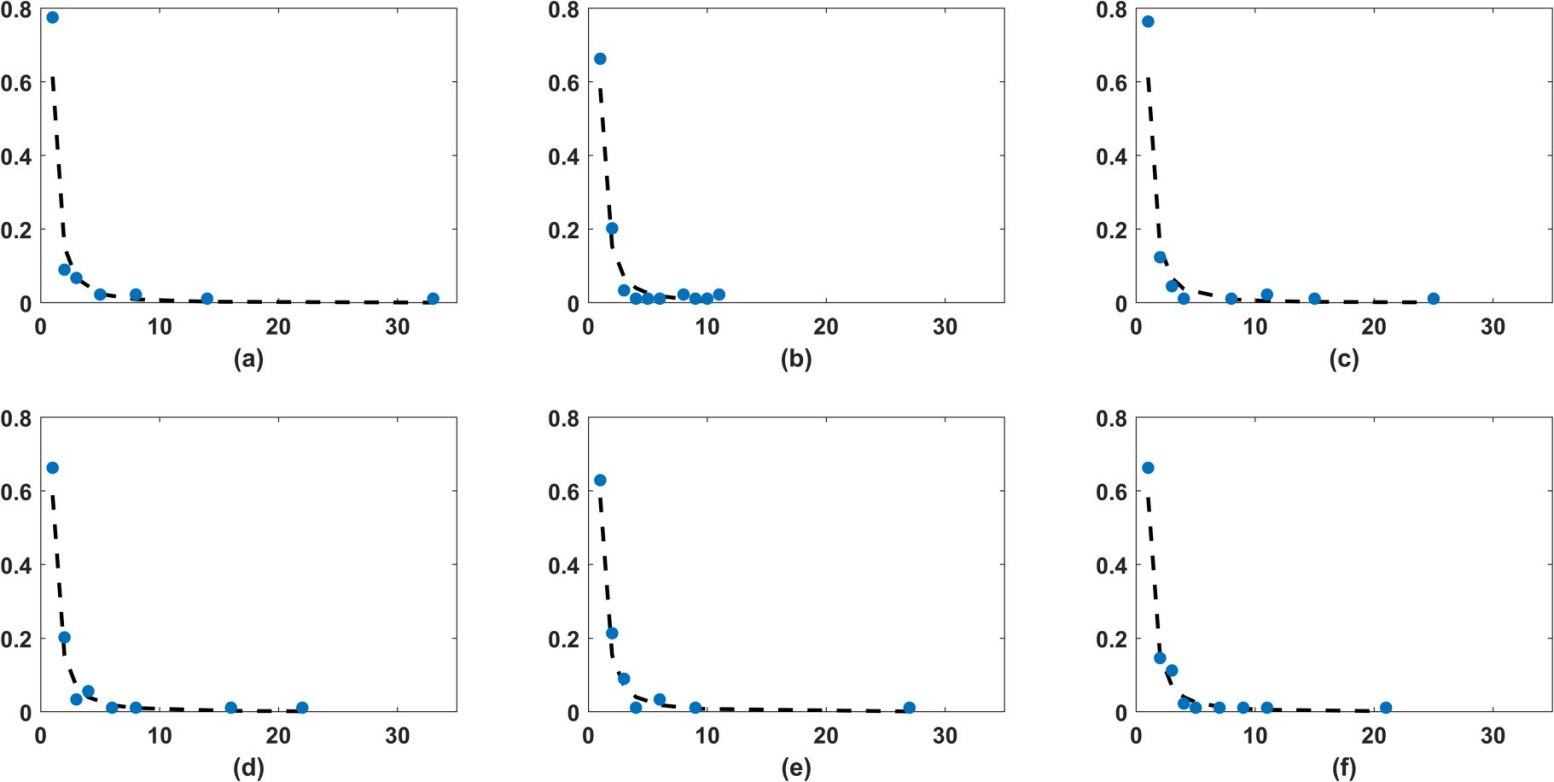
Power law fit to the degree distribution based on mutual information and correlation coefficients. (a) corresponds to network based on mutual information for the pre-election period, (b) corresponds to network based on mutual information for the election period, (c) corresponds to network based on mutual information for the post-election period, similarly (c), (d) and (e) corresponds to network based on correlation coefficient for the pre-election, election and post-election period respectively. Solid blue circles corresponds to empirical frequency and dashed line corresponds to the pdf of fitted power-law distribution.

Emergence of hubs in a network is seen as a property of a scale free network, i.e. a network whose degree distribution follows power law distribution, with power law exponent *α*, 2<*α*<3. We, therefore analyse the probability distribution of the degrees of the stocks in the networks based on correlation coefficient method and mutual information method, for the three time periods. Clauset, Shalizi and Newman [30] suggested that in case of a discrete random variable the power law exponent can be approximated by

$$\hat{\alpha }≅1+n{\left(\sum_{i=1}^{n}ln(\frac{d_{i}}{d_{min}-0.5})\right)}^{-1}$$ (12)

where *n* is number of sample points *d*_1_,*d*_2_,… *d*_*n*_. *d*_*min*_ is the cut-off parameter. Since degrees are always greater than or equal to 1, so we took *d*_*min*_ to be 1 in all the cases. We also used goodness of fit test based on Kolmogrov-Smirnov (KS) statistic to find the *p* -values in order to test if the fit is good or not. A high *p*-values suggest that the power law fit, with exponent $\hat{\alpha }$, is a good fit and a lower *p*-values suggest that it is not. Synthetic data was generated 5000 times to calculate *p*-values in each case. In Fig 7 we plot empirical frequency of the degrees and fitted power law corresponding to each of the six networks. Table 4 gives the respective estimates of *α* and the *p*-values. In all the cases, *p*-values are found to be greater than or equal to 2% (>1% level of significance), in some cases even greater than 10%. Thus, we conclude that the power law is a plausible distribution fit to our data. It is quite evident that the estimated value of $\alpha (\hat{\alpha })$ is observed to be smallest during the election time for the both correlation based network as well as mutual information based network. We also observe that the correlation-based networks did not have the scale-free property. However, mutual information based networks corresponding to pre-election and post-election period had the scale-free property. Further, during the election period the power law exponent corresponding to mutual information based network was less than 2. This is an indication of a thicker tail, i.e., there are greater number of nodes with high degree in the MST network. This suggests that there are stronger interactions amongst the stocks during this time. As an implication, we may observe that portfolio diversification would become less effective during this period.

**Table 4:** Estimated power law exponent $\hat{\alpha }$ for degree distribution.

	$\hat{\alpha }(p\;value)$ Network based on correlation coefficient method	$\hat{\alpha }(p\;value)$ Network based on mutual information method
Pre-election Jan-Feb 2014	1.95(0.14)	2.02(0.02)
Election Mar-May 2014	1.93(0.11)	1.93(0.09)
Post-election Jun-Dec 2014	1.93(0.21)	2.01(0.03)

### 4.3 Eigenvector centrality measure

We also consider the eigenvector centrality measure to identify important stocks in terms of flow of information and to identify the surroundings of such stocks. For each node, we define a relative score such that for a node, its connections to high-scoring nodes contribute more towards its score in comparison to all the low scoring nodes in its neighbourhood. The spectra of the adjacency matrix and Laplacian matrix help us to define such a scoring system and identify the neighbourhoods of highly scoring nodes [31–32].

The adjacency matrix of a graph is a *n*×*n* matrix, where *n* is the number of nodes in the graph, and the (*i*,*j*)*th* entry in the adjacency matrix is 1 if there is an edge between node *i* and *j* and it is 0 if there is no such edge. Adjacency matrix is a non-negative matrix and as an application of the Perron-Frobenius theorem, the eigenvector corresponding to the highest eigenvalue, also known as Perron eigenvector, emerges as a good choice for the scoring system that we are looking for [31]. High scoring stocks based on Perron eigenvector corresponding to mutual information networks for the three timespans, are included as supplementary tables; S1–S3 Tables.

In order to capture the stocks present in the neighbourhood of the stocks with high scores, we consider the Fiedler vector. Fiedler vector is the eigenvector corresponding to the second smallest eigenvalue of the Laplacian matrix [32]. This vector is used to detect communities within the network. We study communities corresponding to high scoring nodes, i.e. the stocks found in the neighbourhood of these high scoring nodes.

In a community, high scoring nodes can be interpreted as key agents, through which information gets transferred to other members in their respective communities. Thus, these high scoring nodes can be thought of as stocks which are more susceptible to market risk at least in comparison to the nodes present in the periphery of their community. Thus, stocks corresponding to the peripheral nodes could act as a good choice for portfolio selection. Pozzi, Matteo and Aste [25] in 2013 suggested a correlation based peripheral stock methodology for portfolio selection. We therefore give a mutual information based peripheral stock methodology and perform a comparative study for the portfolio selection.

### 4.4 Portfolio selection at a high frequency level: a comparative study

**Fig 8:**
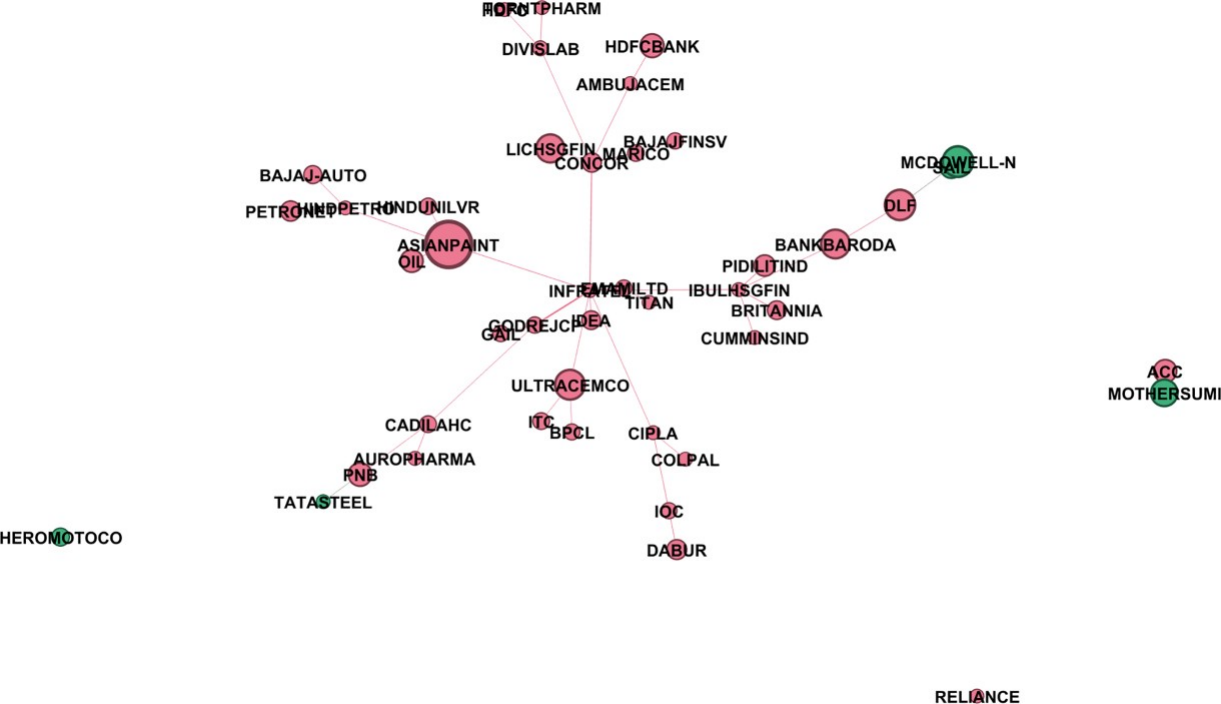
An example of a peripheral portfolio obtained from MI based MST network of 44 stocks. Minimum spanning tree network based on mutual information method for data corresponding to Jan 1, 2014, 9:30:00 hrs to 11:30:00 hours (240 thirty-second time ticks). 5 peripheral stocks out of 44 stocks obtain using method 5 is represented by green colour. Size of nodes are proportional to their corresponding weights in Markowitz portfolio constructed by minimizing the Sharpe ratio.

For an intraday trader it is almost impossible to invest in a large number of stocks. We propose a method where an investor may invest in fewer numbers of stocks and remain competitive with a fully diversified portfolio consisting of larger number of stocks. We worked on 30-second data of 89 stocks picked from CNX100 index of National Stock Exchange of India for the entire year 2014. At each instant, we considered past 2-hours sample data and tested the stability of the portfolios obtained over the next two hours. Firstly, we filtered out the best 45 efficient stocks out of 89 stocks based on high $\bar{r}/s$ ratio, i.e. high ration of sample mean to sample standard deviation, based on the past 2 hours. High $\bar{r}/s$ of a stock ensures that the average return of the stock is high with low standard deviation, thus stock is stable with high returns. These 45 stocks were then further analysed to obtain portfolios based on five different methods:

- Method 1:Markowitz portfolio selection, portfolio which maximizes the Sharpe ratio by investing in all 45 stocks
- Method 2: Markowitz portfolio selection, portfolio which maximizes the Sharpe ratio by investing in all 45 stocks using exponential weighted correlation coefficients [25, 33].
- Method 3: Picking peripheral stocks (stocks with low eigenvector centrality scores) in MST constructed based on correlation method. We analysed portfolios composed of 3, 5, or 10 most peripheral stocks.
- Method 4: Picking peripheral stocks (stocks with low eigenvector centrality scores) in MST constructed based on exponential weighted correlation method [25, 33]. Again, we analysed portfolios composed of 3, 5, or 10 most peripheral stocks.
- Method 5: Picking peripheral stocks (stocks with low eigenvector centrality scores) in MST constructed based on mutual information method. Again, portfolios composed of 3, 5, or 10 most peripheral stocks. Fig 8 corresponds to one such composition of the portfolio which consist of 5 peripheral stocks.

Exponential weighted correlation is calculated using exponential weighted sample mean and exponential weighted sample variance [33] where weights are such that more weight is given to the recent most event in comparison to the past events. Pozzi, Matteo and Aste [33] have suggested the use of exponential weighted correlation based models instead of simple correlation. We thus compare our proposed model with both these models.

**Fig 9:**
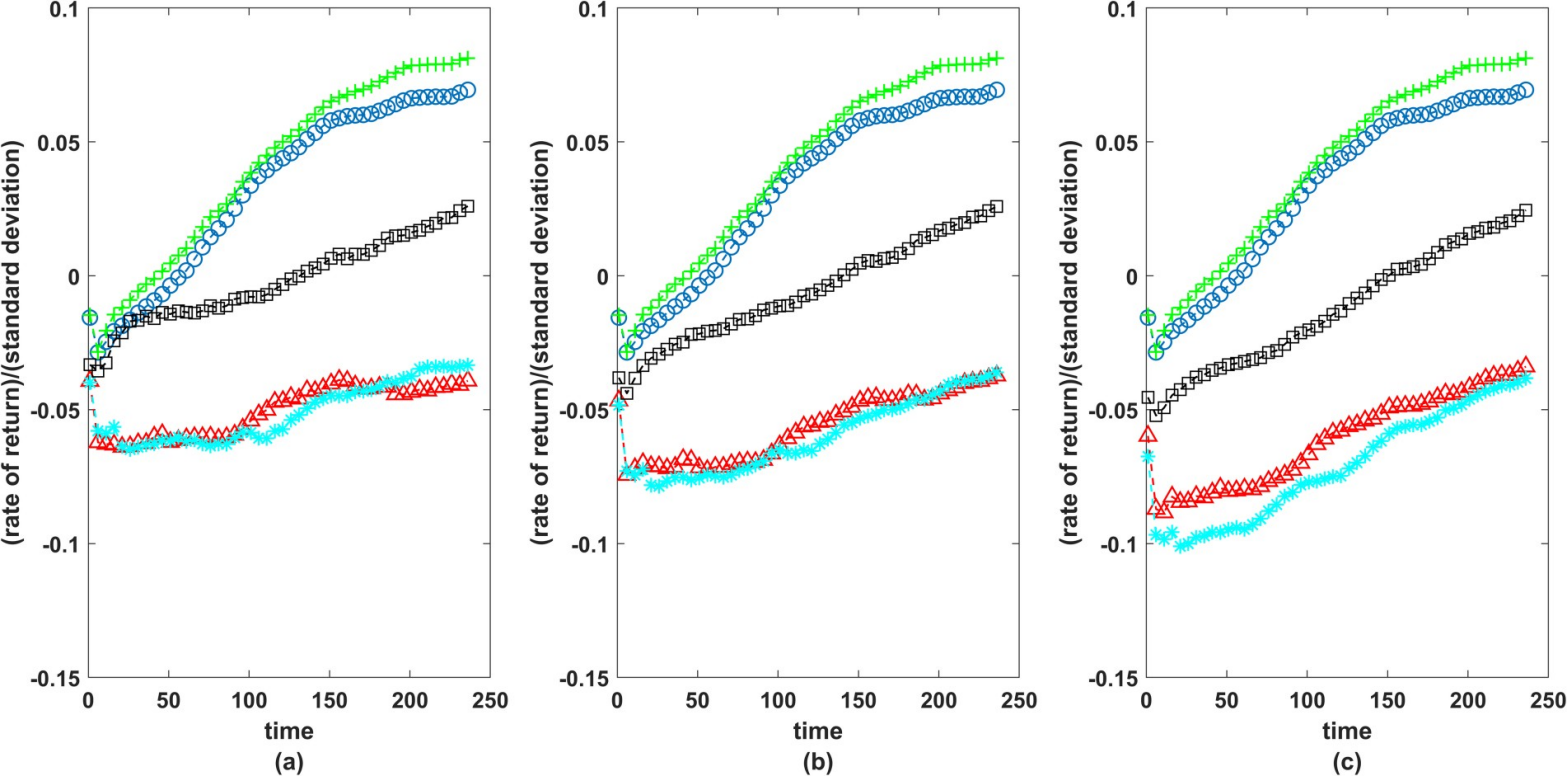
Performance of portfolios. On y-axis we have taken $\bar{r}/s$ (averaged over all 47718 portfolios ~ 1 year, 2014) and on *x*-axis 240 time ticks corresponding to 30second, 1 minute, 1.5 minute…240 minutes, from the time of investments. Green colour plus (+): Markowitz portfolio consisting of 45 stocks which maximizes Sharpe ratio using exponential weighted correlation; Blue colour circle (o): Markowitz portfolio consisting of 45 stocks which maximizes Sharpe ratio using simple correlations; Black colour square (∎): mutual information based portfolio consisting of 5 peripheral stocks; Red colour triangle (⊿): correlation based portfolio consisting of 5 peripheral stocks; Cyan colour cross (*): exponential weighted correlation based portfolio consisting of peripheral stocks; (a) comparison based on 3 peripheral stocks (b) comparison based on 5 peripheral stocks (c) comparison based on 10 peripheral stocks.

At each instant, we took past 2-hour data, constructed the portfolios based on all five methods, a total of 5×47718 portfolios in 1 year (2014), and then tested their performance over the next 2 hours by calculating the respective rate of returns. For each method, there were 47718 such portfolios, and for each portfolio, we calculated the rate of return in the next 30second, 1 minute, 1.5 minute…2 hours from the time of investment. One would like to invest such that the average return $\bar{r}$ is large and at the same time fluctuations are small, i.e. sample standard deviation *s* is small. Thus we tested the performance of each method by calculating $\bar{r}/s$ respectively for the next 30second, 1 minute, 1.5 minute,…2 hour, averaged over all 47718 portfolios. In Fig 9, 240 instants of time corresponding to 2 hours from the time of investment are taken on the *x axis* and $\bar{r}/s$ (averaged over all 47718 portfolios in the entire year) on *y axis*. Clearly, portfolios corresponding to Markowitz method consisting of all 45 stocks perform the best though there is hardly any difference between the case of simple correlations and the case of exponential weighted correlations. From the perspective of an intraday trader, it is almost impossible for a trader to invest in all 45 stocks at a time and thus one would like to invest in best 4 to 6 diversified stocks. Pozzi, Matteo and Aste [25] had earlier suggested to invest in peripheral stocks in the MST network constructed using exponential weighted correlation. We therefore construct portfolios constituting 3, 5 or 10 most peripheral stocks found in the MST networks i.e. stocks with the lowest eigenvector centrality score, corresponding to MST based on correlation, exponential weighted correlation and mutual information methods. Portfolios obtained using mutual information method are seen to be much better in terms of stability and efficiency in comparison to the ones obtained using correlations (Fig 9).

## 5) Conclusion

The aim of this paper was to study interactions between the stocks at a high frequency level with respect to the Indian stock market. For this purpose, we picked 30 seconds as our tick size and studied behaviour of 89 more frequently traded stocks out of 100 stocks listed in CNX100 for the year 2014. We analysed the spectrum of the correlation matrix to study the randomness. More than 40% deviations were observed from the RMT indicating that the pairwise correlation coefficients are not random. We then compared the pairwise correlation coefficients with their respective mutual information. Our analysis showed existence of non-linearity in the high frequency data, that mutual information managed to capture very well. We thus propose that networks based on mutual information, in comparison to the networks based on correlation coefficient, capture the dynamics between the stocks at a high frequency level effectively. Networks constructed using mutual information showed a scale-free property with power law exponent $2<\hat{\alpha }<3$ (*p*–value ≥0.02) in comparison to correlation coefficient method during pre-election and post-election periods. During election period however, power law exponent $\hat{\alpha }$ was observed to be less than 2, indicating that there are quite a number of stocks with large degrees. Thus, a major political event like a general election induces a co-movement of stocks in the market. Also, as a practical application, we have presented a mutual information based methodology of selecting a portfolio consisting of very small number of stocks, as small as 3, 5 or 10, which on an average promises to deliver good returns along with lesser fluctuations even at high frequency level.

Based on our analysis, we finally conclude that stock networks based on the mutual information method capture the dynamics of the stock market more effectively at high frequency level. In future, one may explore the applications of mutual information and other nonlinearity measures to estimate risk more effectively and develop better trading strategies. We also plan to investigate the use of other centrality measures for portfolio selection.

## Supporting information

S1 Fig Log rate of return graphs for CNX100. (a) corresponds to pre-election period, (b) election period and (c) post-election period; x-axis corresponds to tick points and on y-axis we have log rate of returns corresponding to each tick.
(TIF)

S2 Fig Eigenvalue distribution of correlation matrix. (a) pre-election, (b) election and (c) post-election period. Histograms corresponds to empirical probability distribution and solid red line corresponds to the theoretical pdf.
(TIF)

S3 Fig Eigenvalue distribution of correlation matrix on the testing data obtained after reshuffling of the returns of each stock. (a), (b) and (c) are graphs corresponding to pre-election, election and post-election period by testing on one such shuffled dataset. (d), (e) and (f) are graphs corresponding to pre-election, election and post-election period on ensemble testing datasets, i.e. repeating one such trial 50 times. Histograms correspond to empirical probability distribution and solid line corresponds to the theoretical pdf.
(TIF)

S4 Fig Composition of first three principal eigenvectors of the correlation matrix. Bars represents eigenvector components for each stock corresponding to three largest eigenvalues for the three timespan. (a), (d) and (g) corresponds to the largest eigenvector, (b), (e) and (h) corresponds to second largest eigenvector and (c), (f) and (i) corresponds to the third eigenvector for pre-election, election and post-election period respectively. Stocks on the x-axis are arranged according to sectors, A:automobile, B:Consumer Goods, C:Pharmasuticals, D:Financial Services, E:Energy and F:IT sector.
(TIF)

S5 Fig Correlation based MST network, pre-election period (Jan-Feb 2014). Different colours represent different sectors, also size of a node is proportional to the degree of the node and width of the edge is inversely proportional to the distance between two nodes.
(TIF)

S6 Fig Correlation based MST network, election period (Mar-May 2014). Different colours represent different sectors, also size of a node is proportional to the degree of the node and width of the edge is inversely proportional to the distance between two nodes.
(TIF)

S7 Fig Correlation based MST network, post-election period (Jun-Dec 2014). Different colours represent different sectors, also size of a node is proportional to the degree of the node and width of the edge is inversely proportional to the distance between two nodes.
(TIF)

S1 Table High scoring stocks with scores from Perron vector, pre-election period (Jan-Feb 2014). (DOCX)

S2 Table High scoring stocks with scores from Perron vector, election period (Mar-May 2014). (DOCX)

S3 Table High scoring stocks with scores from Perron vector, post-election period (Jun-Dec 2014). (DOCX)

S4 Table Data corresponding to Figs 1–9, S1–S7 Figs. (XLSX)
