# Supplementary figures and images for: Mutual information based stock networks and portfolio selection for intraday traders using high frequency data: An Indian market case study
Source: PLoS One. 2019 Aug 29;14(8):e0221910. doi: 10.1371/journal.pone.0221910 (PMC6715228; doi:10.1371/journal.pone.0221910)

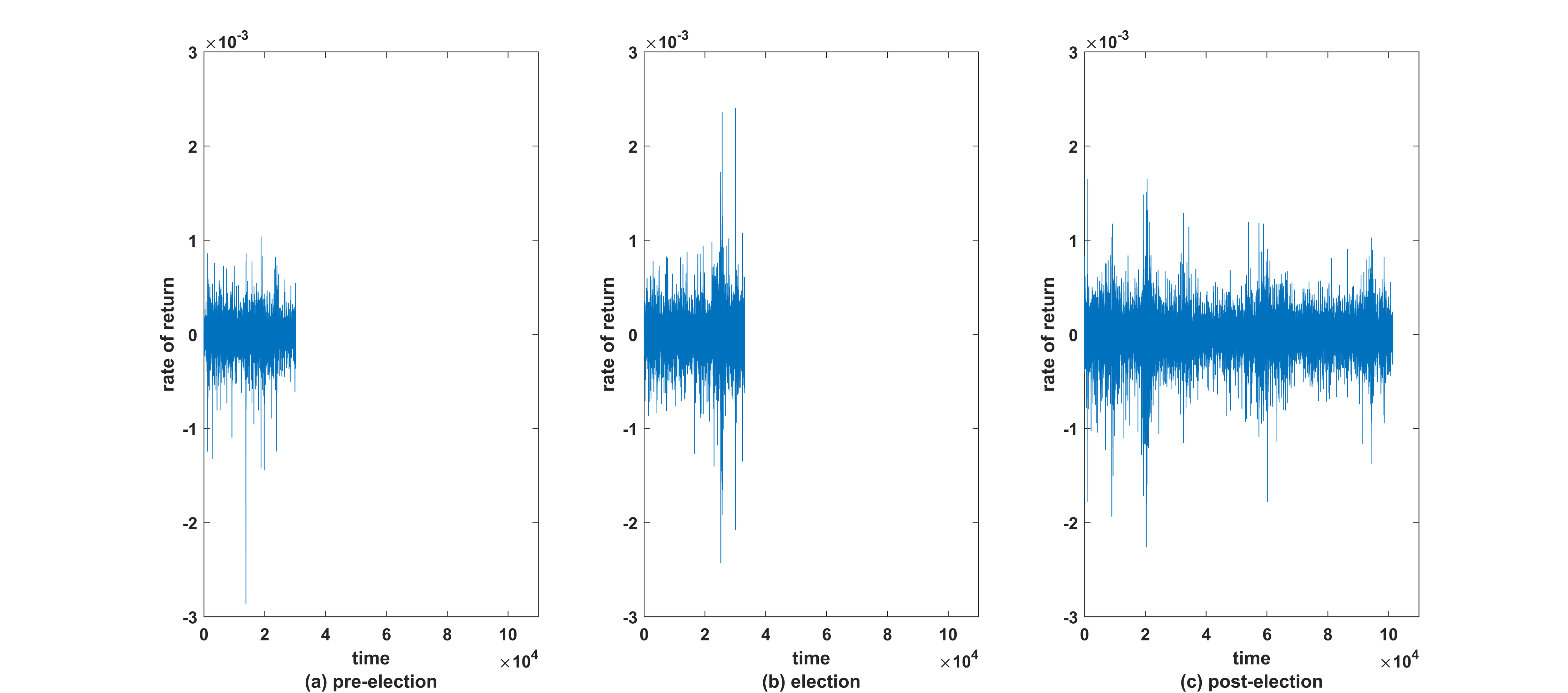

Supplement: S1 Fig — (a) corresponds to pre-election period, (b) election period and (c) post-election period; x-axis corresponds to tick points and on y-axis we have log rate of returns corresponding to each tick. (TIF) [file pone.0221910.s001.tif]

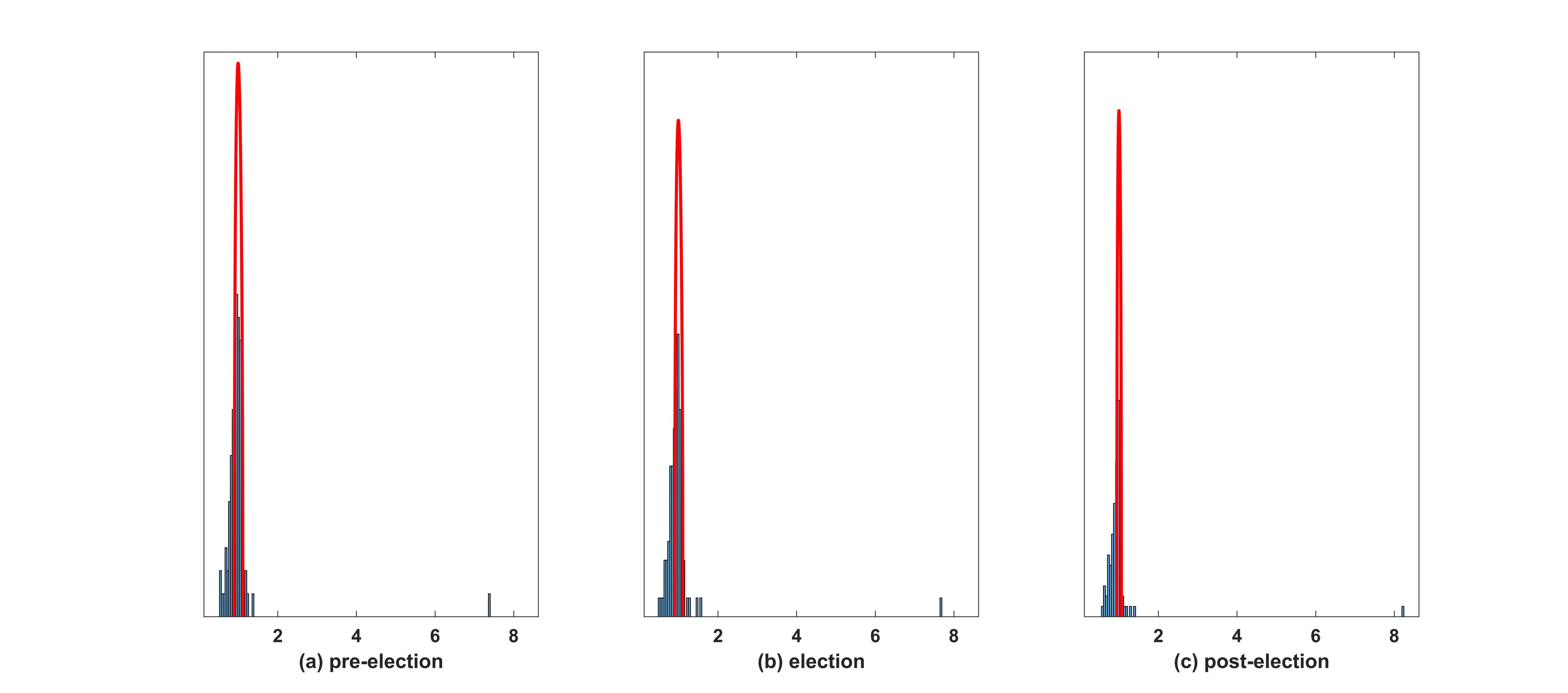

Supplement: S2 Fig — (a) pre-election, (b) election and (c) post-election period. Histograms corresponds to empirical probability distribution and solid red line corresponds to the theoretical pdf. (TIF) [file pone.0221910.s002.tif]

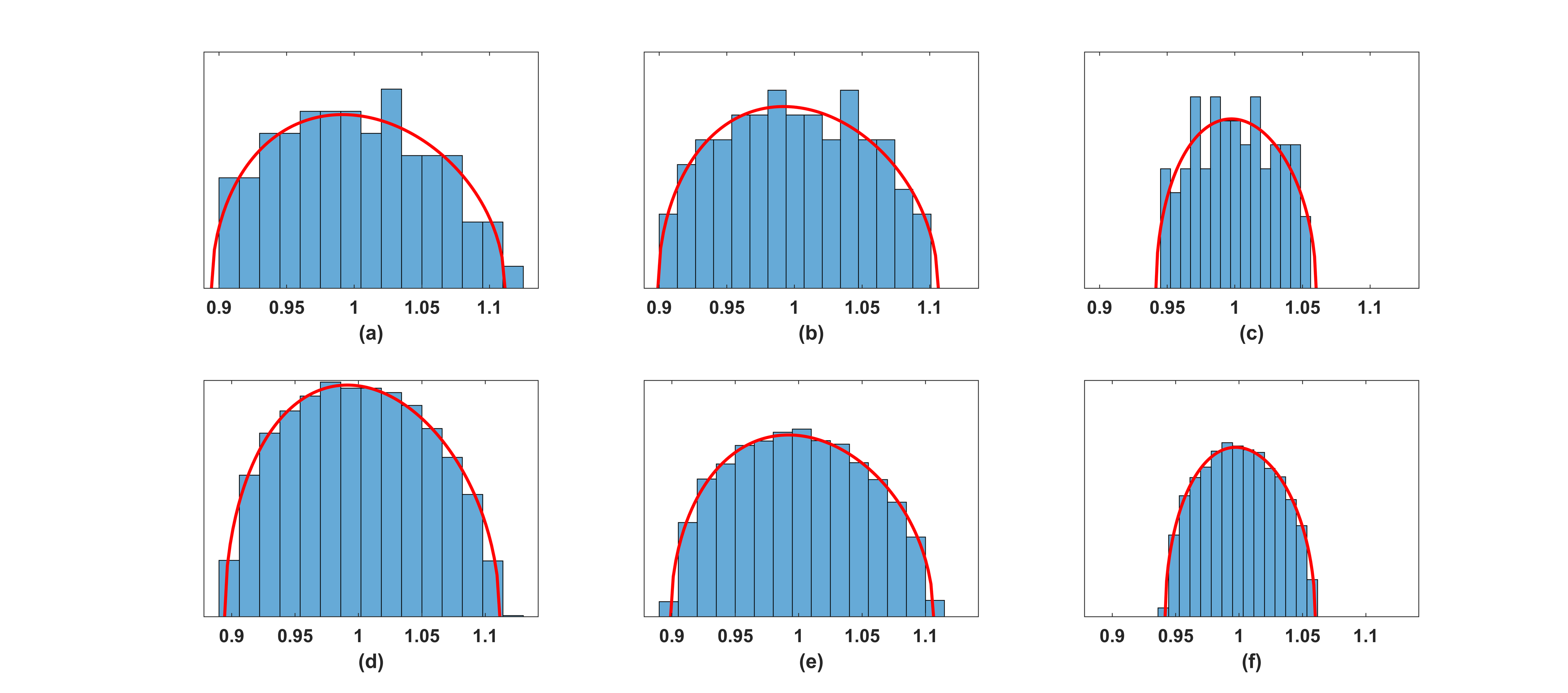

Supplement: S3 Fig — (a), (b) and (c) are graphs corresponding to pre-election, election and post-election period by testing on one such shuffled dataset. (d), (e) and (f) are graphs corresponding to pre-election, election and post-election period on ensemble testing datasets, i.e. repeating one such trial 50 times. Histograms correspond to empirical probability distribution and solid line corresponds to the theoretical pdf. (TIF) [file pone.0221910.s003.tif]

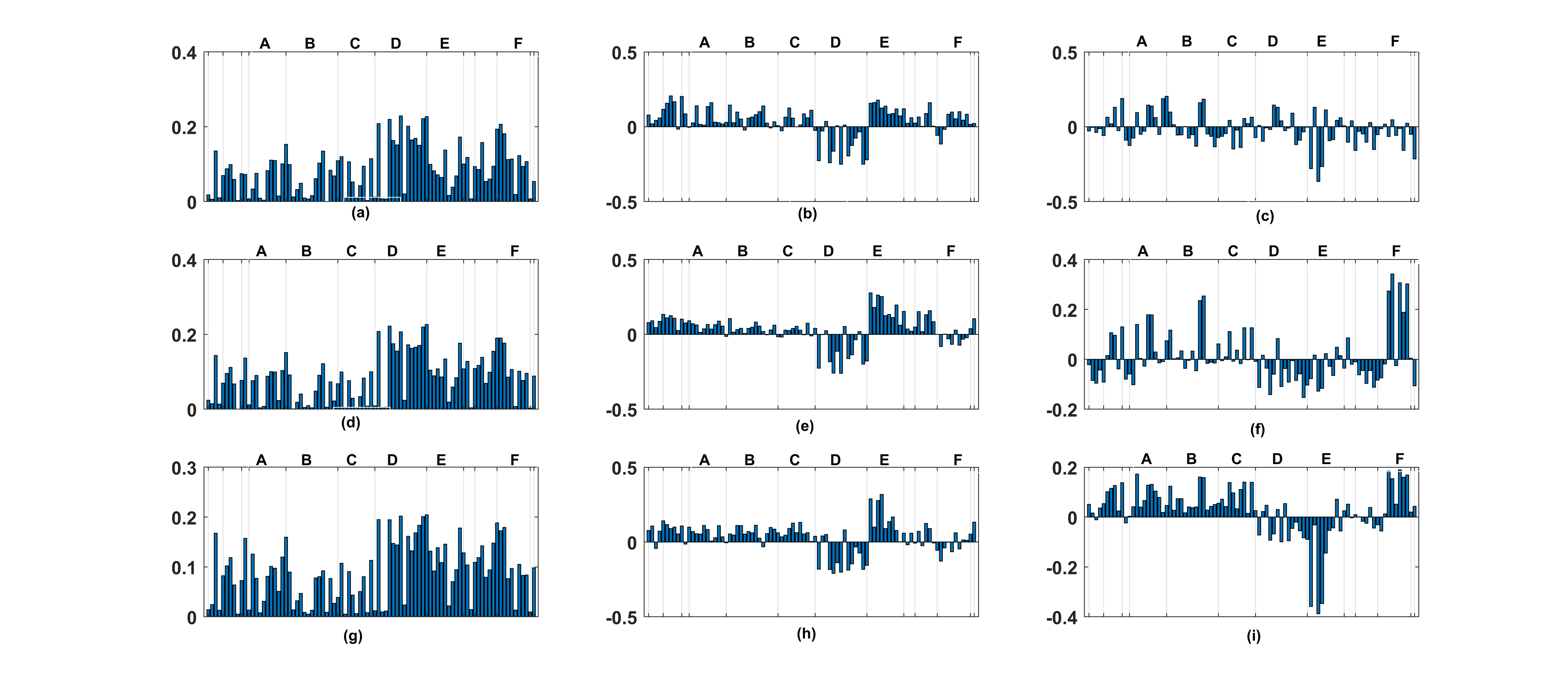

Supplement: S4 Fig — Bars represents eigenvector components for each stock corresponding to three largest eigenvalues for the three timespan. (a), (d) and (g) corresponds to the largest eigenvector, (b), (e) and (h) corresponds to second largest eigenvector and (c), (f) and (i) corresponds to the third eigenvector for pre-election, election and post-election period respectively. Stocks on the x-axis are arranged according to sectors, A:automobile, B:Consumer Goods, C:Pharmasuticals, D:Financial Services, E:Energy and F:IT sector. (TIF) [file pone.0221910.s004.tif]

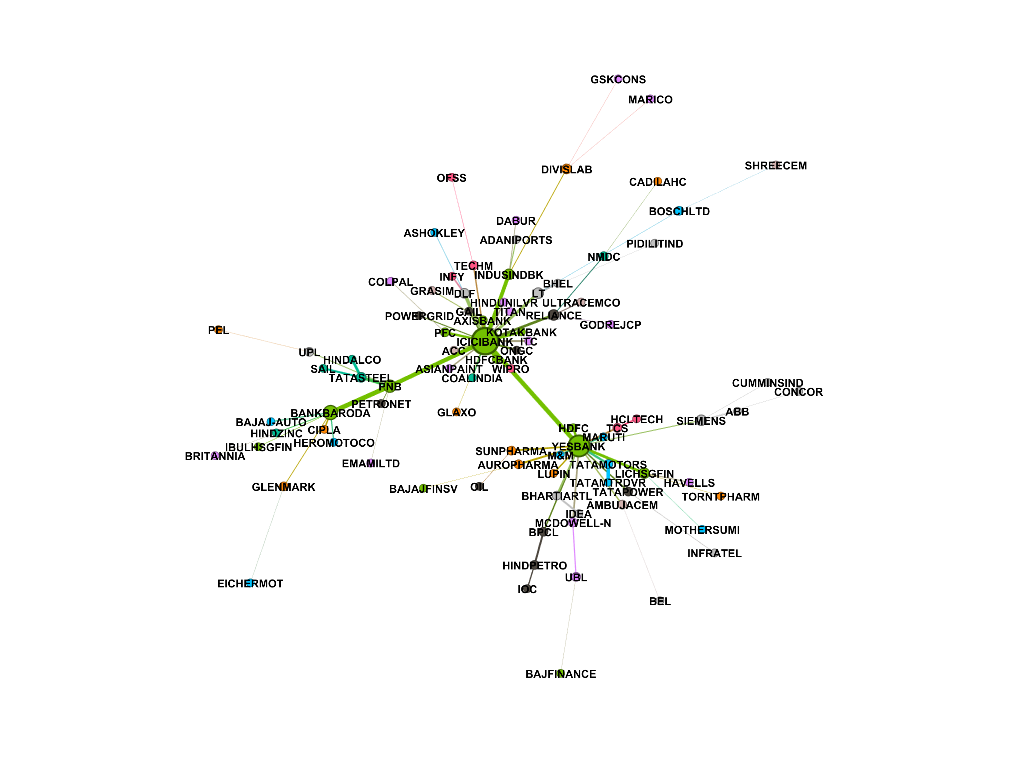

Supplement: S5 Fig — Different colours represent different sectors, also size of a node is proportional to the degree of the node and width of the edge is inversely proportional to the distance between two nodes. (TIF) [file pone.0221910.s005.tif]

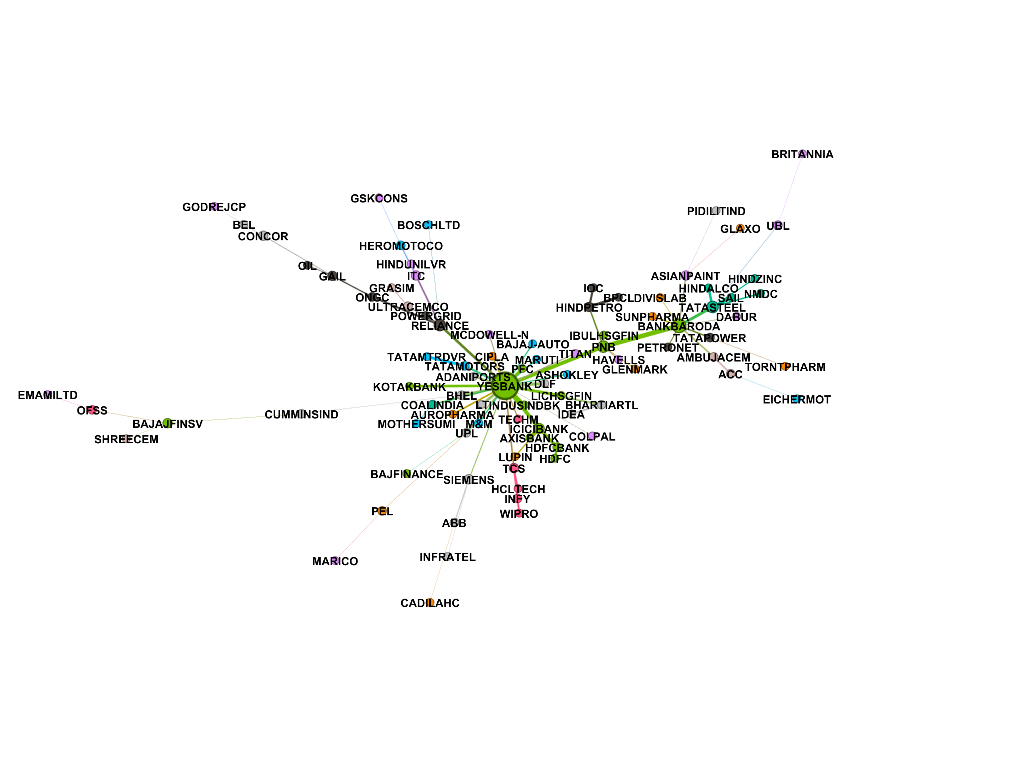

Supplement: S6 Fig — Different colours represent different sectors, also size of a node is proportional to the degree of the node and width of the edge is inversely proportional to the distance between two nodes. (TIF) [file pone.0221910.s006.tif]

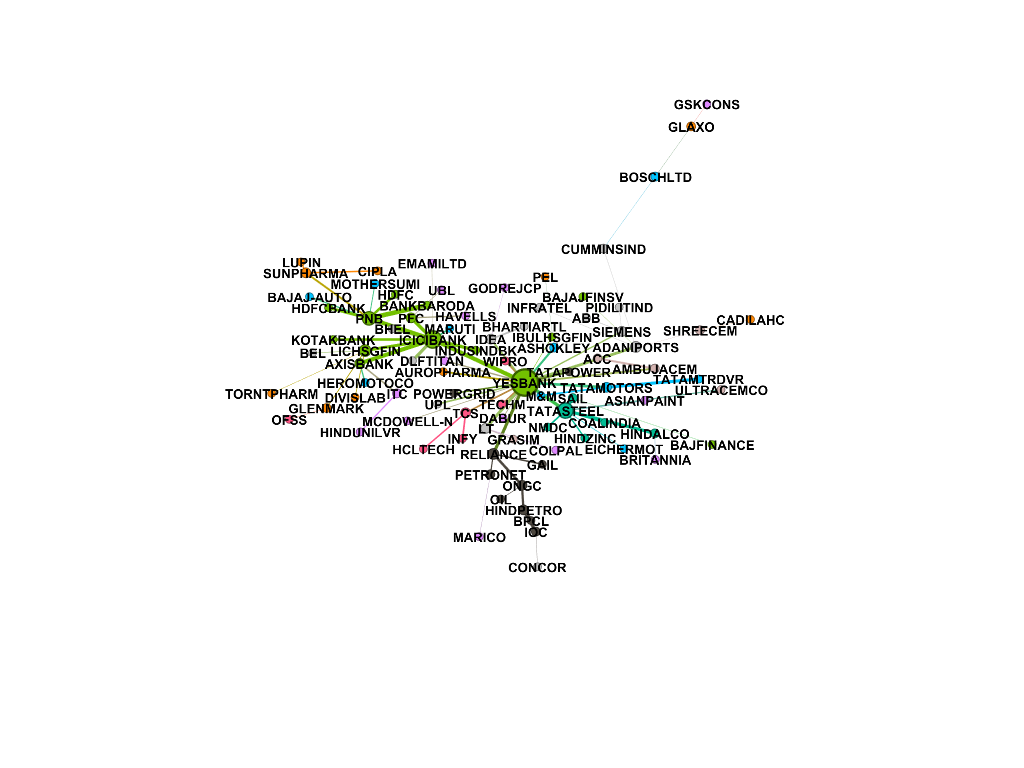

Supplement: S7 Fig — Different colours represent different sectors, also size of a node is proportional to the degree of the node and width of the edge is inversely proportional to the distance between two nodes. (TIF) [file pone.0221910.s007.tif]
