# Supplementary material for: Mutual information based stock networks and portfolio selection for intraday traders using high frequency data: An Indian market case study
Source: PLoS One. 2019 Aug 29;14(8):e0221910. doi: 10.1371/journal.pone.0221910 (PMC6715228; doi:10.1371/journal.pone.0221910)
Supplement: S1 Table — (DOCX) [file pone.0221910.s008.docx]

**S1 Table: High scoring stocks with scores from Perron vector, for the pre- election period i.e. Jan-Feb2014**

| High scoring stocks with scores from Perron vector , Jan-Feb 2014 | | | | | |
| --- | --- | --- | --- | --- | --- |
| correlation method | | | mutual information | | |
| Name | Business Sector | normalized score in eigenvector corresponding to largest eigenvalue | Name | Business Sector | normalized score in eigenvector corresponding to largest eigenvalue |
| ICICIBANK | FINANCIAL SERVICES | 10.85% | CONCOR | SERVICES | 14.19% |
| YESBANK | FINANCIAL SERVICES | 6.01% | BEL | INDUSTRIAL MANUFACTURING | 2.80% |
| PNB | FINANCIAL SERVICES | 2.81% | HINDPETRO | ENERGY | 2.62% |
| INDUSINDBK | FINANCIAL SERVICES | 2.50% | TATAMTRDVR | AUTOMOBILE | 2.54% |
| LT | CONSTRUCTION | 2.49% | BAJFINANCE | FINANCIAL SERVICES | 2.54% |
| RELIANCE | ENERGY | 2.49% | ABB | INDUSTRIAL MANUFACTURING | 2.46% |
|  |  |  | ACC | CEMENT & CEMENT PRODUCTS | 2.46% |
|  |  |  | ADANIPORTS | SERVICES | 2.46% |
|  |  |  | BAJAJFINSV | FINANCIAL SERVICES | 2.46% |
|  |  |  | INFRATEL | TELECOM | 2.46% |
|  |  |  | BOSCHLTD | AUTOMOBILE | 2.46% |
|  |  |  | BRITANNIA | CONSUMER GOODS | 2.46% |
|  |  |  | CADILAHC | PHARMA | 2.46% |
|  |  |  | CUMMINSIND | INDUSTRIAL MANUFACTURING | 2.46% |
|  |  |  | DIVISLAB | PHARMA | 2.46% |
|  |  |  | EICHERMOT | AUTOMOBILE | 2.46% |
|  |  |  | EMAMILTD | CONSUMER GOODS | 2.46% |
|  |  |  | GSKCONS | CONSUMER GOODS | 2.46% |
|  |  |  | GLAXO | PHARMA | 2.46% |
