# Supplementary material for: Mutual information based stock networks and portfolio selection for intraday traders using high frequency data: An Indian market case study
Source: PLoS One. 2019 Aug 29;14(8):e0221910. doi: 10.1371/journal.pone.0221910 (PMC6715228; doi:10.1371/journal.pone.0221910)
Supplement: S2 Table — (DOCX) [file pone.0221910.s009.docx]

**S2 Table: High scoring stocks with scores from Perron vector, for the election period i.e. Mar-May 2014**

| High scoring stocks with scores from Perron vector, Mar-May 2014 | | | | | |
| --- | --- | --- | --- | --- | --- |
| correlation method | | | mutual information | | |
| Name | Business Sector | normalized score in eigenvector corresponding to largest eigenvalue | Name | Business Sector | normalized score in eigenvector corresponding to largest eigenvalue |
| YESBANK | FINANCIAL SERVICES | 13.59% | PNB | FINANCIAL SERVICES | 9.63% |
| PNB | FINANCIAL SERVICES | 3.13% | YESBANK | FINANCIAL SERVICES | 6.96% |
| RELIANCE | ENERGY | 3.06% | BANKBARODA | FINANCIAL SERVICES | 6.36% |
| ICICIBANK | FINANCIAL SERVICES | 2.90% | TATASTEEL | METALS | 3.45% |
| TCS | IT | 2.78% | RELIANCE | ENERGY | 3.18% |
| BHEL | INDUSTRIAL MANUFACTURING | 2.68% | BHARTIARTL | TELECOM | 2.68% |
| BHARTIARTL | TELECOM | 2.68% | BHEL | INDUSTRIAL MANUFACTURING | 2.67% |
| TATAMOTORS | AUTOMOBILE | 2.68% | LT | CONSTRUCTION | 2.67% |
| TECHM | IT | 2.68% |  |  |  |
| ADANIPORTS | SERVICES | 2.58% |  |  |  |
| ASHOKLEY | AUTOMOBILE | 2.58% |  |  |  |
| AUROPHARMA | PHARMA | 2.58% |  |  |  |
| BAJAJ-AUTO | AUTOMOBILE | 2.58% |  |  |  |
| CIPLA | PHARMA | 2.58% |  |  |  |
| COALINDIA | METALS | 2.58% |  |  |  |
| COLPAL | CONSUMER GOODS | 2.58% |  |  |  |
| DLF | CONSTRUCTION | 2.58% |  |  |  |
| HAVELLS | CONSUMER GOODS | 2.58% |  |  |  |
| INDUSINDBK | FINANCIAL SERVICES | 2.58% |  |  |  |
| KOTAKBANK | FINANCIAL SERVICES | 2.58% |  |  |  |
| LICHSGFIN | FINANCIAL SERVICES | 2.58% |  |  |  |
| LT | CONSTRUCTION | 2.58% |  |  |  |
| M&M | AUTOMOBILE | 2.58% |  |  |  |
| MARUTI | AUTOMOBILE | 2.58% |  |  |  |
| PFC | FINANCIAL SERVICES | 2.58% |  |  |  |
| TITAN | CONSUMER GOODS | 2.58% |  |  |  |
| MCDOWELL-N | CONSUMER GOODS | 2.58% |  |  |  |
