# Supplementary material for: Mutual information based stock networks and portfolio selection for intraday traders using high frequency data: An Indian market case study
Source: PLoS One. 2019 Aug 29;14(8):e0221910. doi: 10.1371/journal.pone.0221910 (PMC6715228; doi:10.1371/journal.pone.0221910)
Supplement: S3 Table — (DOCX) [file pone.0221910.s010.docx]

**S3 Table: High scoring stocks with scores from Perron vector, for the post-election period i.e. Jun-Dec 2014**

| High scoring stocks with scores from Perron vector Jun-Dec 2014 | | | | | |
| --- | --- | --- | --- | --- | --- |
| correlation method | | | mutual information | | |
| Name | Business Sector | normalized score in eigenvector corresponding to largest eigenvalue | Name | Business Sector | normalized score in eigenvector corresponding to largest eigenvalue |
| YESBANK | FINANCIAL SERVICES | 11.78% | GLAXO | PHARMA | 15.44% |
| ICICIBANK | FINANCIAL SERVICES | 4.54% | GRASIM | CEMENT & CEMENT PRODUCTS | 3.33% |
| TATASTEEL | METALS | 3.74% | ABB | INDUSTRIAL MANUFACTURING | 3.08% |
| RELIANCE | ENERGY | 2.82% | BAJFINANCE | FINANCIAL SERVICES | 3.08% |
| ADANIPORTS | SERVICES | 2.81% | BAJAJFINSV | FINANCIAL SERVICES | 3.08% |
| IDEA | TELECOM | 2.67% | BEL | INDUSTRIAL MANUFACTURING | 3.08% |
| LT | CONSTRUCTION | 2.67% | INFRATEL | TELECOM | 3.08% |
| SIEMENS | INDUSTRIAL MANUFACTURING | 2.67% | BOSCHLTD | AUTOMOBILE | 3.08% |
| TCS | IT | 2.67% | BRITANNIA | CONSUMER GOODS | 3.08% |
| TATAMOTORS | AUTOMOBILE | 2.67% | CADILAHC | PHARMA | 3.08% |
| AMBUJACEM | CEMENT & CEMENT PRODUCTS | 2.56% | COLPAL | CONSUMER GOODS | 3.08% |
| ASHOKLEY | AUTOMOBILE | 2.55% | CONCOR | SERVICES | 3.08% |
| AUROPHARMA | PHARMA | 2.44% | CUMMINSIND | INDUSTRIAL MANUFACTURING | 3.08% |
| DABUR | CONSUMER GOODS | 2.44% | EICHERMOT | AUTOMOBILE | 3.08% |
| IBULHSGFIN | FINANCIAL SERVICES | 2.44% | EMAMILTD | CONSUMER GOODS | 3.08% |
| POWERGRID | ENERGY | 2.44% | GSKCONS | CONSUMER GOODS | 3.08% |
| TATAPOWER | ENERGY | 2.44% | GODREJCP | CONSUMER GOODS | 3.08% |
| TECHM | IT | 2.44% | IBULHSGFIN | FINANCIAL SERVICES | 3.08% |
| TITAN | CONSUMER GOODS | 2.44% | MARICO | CONSUMER GOODS | 3.08% |
| UPL | FERTILISERS & PESTICIDES | 2.44% | OIL | ENERGY | 3.08% |
| MCDOWELL-N | CONSUMER GOODS | 2.44% | OFSS | IT | 3.08% |
| WIPRO | IT | 2.44% | PIDILITIND | CHEMICALS | 3.08% |
|  |  |  | PEL | PHARMA | 3.08% |
|  |  |  | SHREECEM | CEMENT & CEMENT PRODUCTS | 3.08% |
|  |  |  | TORNTPHARM | PHARMA | 3.08% |
|  |  |  | UBL | CONSUMER GOODS | 3.08% |
